# Energy dependence of forward-rapidity $\mathrm {J}/\psi$ and $\psi \mathrm {(2S)}$ production in pp collisions at the LHC

**DOI:** 10.1140/epjc/s10052-017-4940-4

**Published:** 2017-06-14

**Authors:** S. Acharya, D. Adamová, M. M. Aggarwal, G. Aglieri Rinella, M. Agnello, N. Agrawal, Z. Ahammed, N. Ahmad, S. U. Ahn, S. Aiola, A. Akindinov, S. N. Alam, D. S. D. Albuquerque, D. Aleksandrov, B. Alessandro, D. Alexandre, R. Alfaro Molina, A. Alici, A. Alkin, J. Alme, T. Alt, I. Altsybeev, C. Alves Garcia Prado, M. An, C. Andrei, H. A. Andrews, A. Andronic, V. Anguelov, C. Anson, T. Antičić, F. Antinori, P. Antonioli, R. Anwar, L. Aphecetche, H. Appelshäuser, S. Arcelli, R. Arnaldi, O. W. Arnold, I. C. Arsene, M. Arslandok, B. Audurier, A. Augustinus, R. Averbeck, M. D. Azmi, A. Badalà, Y. W. Baek, S. Bagnasco, R. Bailhache, R. Bala, A. Baldisseri, M. Ball, R. C. Baral, A. M. Barbano, R. Barbera, F. Barile, L. Barioglio, G. G. Barnaföldi, L. S. Barnby, V. Barret, P. Bartalini, K. Barth, J. Bartke, E. Bartsch, M. Basile, N. Bastid, S. Basu, B. Bathen, G. Batigne, A. Batista Camejo, B. Batyunya, P. C. Batzing, I. G. Bearden, H. Beck, C. Bedda, N. K. Behera, I. Belikov, F. Bellini, H. Bello Martinez, R. Bellwied, L. G. E. Beltran, V. Belyaev, G. Bencedi, S. Beole, A. Bercuci, Y. Berdnikov, D. Berenyi, R. A. Bertens, D. Berzano, L. Betev, A. Bhasin, I. R. Bhat, A. K. Bhati, B. Bhattacharjee, J. Bhom, L. Bianchi, N. Bianchi, C. Bianchin, J. Bielčík, J. Bielčíková, A. Bilandzic, G. Biro, R. Biswas, S. Biswas, J. T. Blair, D. Blau, C. Blume, G. Boca, F. Bock, A. Bogdanov, L. Boldizsár, M. Bombara, G. Bonomi, M. Bonora, J. Book, H. Borel, A. Borissov, M. Borri, E. Botta, C. Bourjau, P. Braun-Munzinger, M. Bregant, T. A. Broker, T. A. Browning, M. Broz, E. J. Brucken, E. Bruna, G. E. Bruno, D. Budnikov, H. Buesching, S. Bufalino, P. Buhler, S. A. I. Buitron, P. Buncic, O. Busch, Z. Buthelezi, J. B. Butt, J. T. Buxton, J. Cabala, D. Caffarri, H. Caines, A. Caliva, E. Calvo Villar, P. Camerini, A. A. Capon, F. Carena, W. Carena, F. Carnesecchi, J. Castillo Castellanos, A. J. Castro, E. A. R. Casula, C. Ceballos Sanchez, P. Cerello, B. Chang, S. Chapeland, M. Chartier, J. L. Charvet, S. Chattopadhyay, S. Chattopadhyay, A. Chauvin, M. Cherney, C. Cheshkov, B. Cheynis, V. Chibante Barroso, D. D. Chinellato, S. Cho, P. Chochula, K. Choi, M. Chojnacki, S. Choudhury, P. Christakoglou, C. H. Christensen, P. Christiansen, T. Chujo, S. U. Chung, C. Cicalo, L. Cifarelli, F. Cindolo, J. Cleymans, F. Colamaria, D. Colella, A. Collu, M. Colocci, M. Concas, G. Conesa Balbastre, Z. Conesa del Valle, M. E. Connors, J. G. Contreras, T. M. Cormier, Y. Corrales Morales, I. Cortés Maldonado, P. Cortese, M. R. Cosentino, F. Costa, S. Costanza, J. Crkovská, P. Crochet, E. Cuautle, L. Cunqueiro, T. Dahms, A. Dainese, M. C. Danisch, A. Danu, D. Das, I. Das, S. Das, A. Dash, S. Dash, S. De, A. De Caro, G. de Cataldo, C. de Conti, J. de Cuveland, A. De Falco, D. De Gruttola, N. De Marco, S. De Pasquale, R. D. De Souza, H. F. Degenhardt, A. Deisting, A. Deloff, C. Deplano, P. Dhankher, D. Di Bari, A. Di Mauro, P. Di Nezza, B. Di Ruzza, M. A. Diaz Corchero, T. Dietel, P. Dillenseger, R. Divià, Ø. Djuvsland, A. Dobrin, D. Domenicis Gimenez, B. Dönigus, O. Dordic, T. Drozhzhova, A. K. Dubey, A. Dubla, L. Ducroux, A. K. Duggal, P. Dupieux, R. J. Ehlers, D. Elia, E. Endress, H. Engel, E. Epple, B. Erazmus, F. Erhardt, B. Espagnon, S. Esumi, G. Eulisse, J. Eum, D. Evans, S. Evdokimov, L. Fabbietti, J. Faivre, A. Fantoni, M. Fasel, L. Feldkamp, A. Feliciello, G. Feofilov, J. Ferencei, A. Fernández Téllez, E. G. Ferreiro, A. Ferretti, A. Festanti, V. J. G. Feuillard, J. Figiel, M. A. S. Figueredo, S. Filchagin, D. Finogeev, F. M. Fionda, E. M. Fiore, M. Floris, S. Foertsch, P. Foka, S. Fokin, E. Fragiacomo, A. Francescon, A. Francisco, U. Frankenfeld, G. G. Fronze, U. Fuchs, C. Furget, A. Furs, M. Fusco Girard, J. J. Gaardhøje, M. Gagliardi, A. M. Gago, K. Gajdosova, M. Gallio, C. D. Galvan, P. Ganoti, C. Gao, C. Garabatos, E. Garcia-Solis, K. Garg, P. Garg, C. Gargiulo, P. Gasik, E. F. Gauger, M. B. Gay Ducati, M. Germain, P. Ghosh, S. K. Ghosh, P. Gianotti, P. Giubellino, P. Giubilato, E. Gladysz-Dziadus, P. Glässel, D. M. Goméz Coral, A. Gomez Ramirez, A. S. Gonzalez, V. Gonzalez, P. González-Zamora, S. Gorbunov, L. Görlich, S. Gotovac, V. Grabski, L. K. Graczykowski, K. L. Graham, L. Greiner, A. Grelli, C. Grigoras, V. Grigoriev, A. Grigoryan, S. Grigoryan, N. Grion, J. M. Gronefeld, F. Grosa, J. F. Grosse-Oetringhaus, R. Grosso, L. Gruber, F. R. Grull, F. Guber, R. Guernane, B. Guerzoni, K. Gulbrandsen, T. Gunji, A. Gupta, R. Gupta, I. B. Guzman, R. Haake, C. Hadjidakis, H. Hamagaki, G. Hamar, J. C. Hamon, J. W. Harris, A. Harton, D. Hatzifotiadou, S. Hayashi, S. T. Heckel, E. Hellbär, H. Helstrup, A. Herghelegiu, G. Herrera Corral, F. Herrmann, B. A. Hess, K. F. Hetland, H. Hillemanns, B. Hippolyte, J. Hladky, B. Hohlweger, D. Horak, R. Hosokawa, P. Hristov, C. Hughes, T. J. Humanic, N. Hussain, T. Hussain, D. Hutter, D. S. Hwang, R. Ilkaev, M. Inaba, M. Ippolitov, M. Irfan, V. Isakov, M. S. Islam, M. Ivanov, V. Ivanov, V. Izucheev, B. Jacak, N. Jacazio, P. M. Jacobs, M. B. Jadhav, S. Jadlovska, J. Jadlovsky, S. Jaelani, C. Jahnke, M. J. Jakubowska, M. A. Janik, P. H. S. Y. Jayarathna, C. Jena, S. Jena, M. Jercic, R. T. Jimenez Bustamante, P. G. Jones, A. Jusko, P. Kalinak, A. Kalweit, J. H. Kang, V. Kaplin, S. Kar, A. Karasu Uysal, O. Karavichev, T. Karavicheva, L. Karayan, E. Karpechev, U. Kebschull, R. Keidel, D. L. D. Keijdener, M. Keil, B. Ketzer, M. Mohisin Khan, P. Khan, S. A. Khan, A. Khanzadeev, Y. Kharlov, A. Khatun, A. Khuntia, M. M. Kielbowicz, B. Kileng, D. Kim, D. W. Kim, D. J. Kim, H. Kim, J. S. Kim, J. Kim, M. Kim, M. Kim, S. Kim, T. Kim, S. Kirsch, I. Kisel, S. Kiselev, A. Kisiel, G. Kiss, J. L. Klay, C. Klein, J. Klein, C. Klein-Bösing, S. Klewin, A. Kluge, M. L. Knichel, A. G. Knospe, C. Kobdaj, M. Kofarago, T. Kollegger, A. Kolojvari, V. Kondratiev, N. Kondratyeva, E. Kondratyuk, A. Konevskikh, M. Kopcik, M. Kour, C. Kouzinopoulos, O. Kovalenko, V. Kovalenko, M. Kowalski, G. Koyithatta Meethaleveedu, I. Králik, A. Kravčáková, M. Krivda, F. Krizek, E. Kryshen, M. Krzewicki, A. M. Kubera, V. Kučera, C. Kuhn, P. G. Kuijer, A. Kumar, J. Kumar, L. Kumar, S. Kumar, S. Kundu, P. Kurashvili, A. Kurepin, A. B. Kurepin, A. Kuryakin, S. Kushpil, M. J. Kweon, Y. Kwon, S. L. La Pointe, P. La Rocca, C. Lagana Fernandes, I. Lakomov, R. Langoy, K. Lapidus, C. Lara, A. Lardeux, A. Lattuca, E. Laudi, R. Lavicka, L. Lazaridis, R. Lea, L. Leardini, S. Lee, F. Lehas, S. Lehner, J. Lehrbach, R. C. Lemmon, V. Lenti, E. Leogrande, I. León Monzón, P. Lévai, S. Li, X. Li, J. Lien, R. Lietava, S. Lindal, V. Lindenstruth, C. Lippmann, M. A. Lisa, V. Litichevskyi, H. M. Ljunggren, W. J. Llope, D. F. Lodato, P. I. Loenne, V. Loginov, C. Loizides, P. Loncar, X. Lopez, E. López Torres, A. Lowe, P. Luettig, M. Lunardon, G. Luparello, M. Lupi, T. H. Lutz, A. Maevskaya, M. Mager, S. Mahajan, S. M. Mahmood, A. Maire, R. D. Majka, M. Malaev, I. Maldonado Cervantes, L. Malinina, D. Mal’Kevich, P. Malzacher, A. Mamonov, V. Manko, F. Manso, V. Manzari, Y. Mao, M. Marchisone, J. Mareš, G. V. Margagliotti, A. Margotti, J. Margutti, A. Marín, C. Markert, M. Marquard, N. A. Martin, P. Martinengo, J. A. L. Martinez, M. I. Martínez, G. Martínez García, M. Martinez Pedreira, A. Mas, S. Masciocchi, M. Masera, A. Masoni, A. Mastroserio, A. M. Mathis, A. Matyja, C. Mayer, J. Mazer, M. Mazzilli, M. A. Mazzoni, F. Meddi, Y. Melikyan, A. Menchaca-Rocha, E. Meninno, J. Mercado Pérez, M. Meres, S. Mhlanga, Y. Miake, M. M. Mieskolainen, D. L. Mihaylov, K. Mikhaylov, L. Milano, J. Milosevic, A. Mischke, A. N. Mishra, D. Miśkowiec, J. Mitra, C. M. Mitu, N. Mohammadi, B. Mohanty, E. Montes, D. A. Moreira De Godoy, L. A. P. Moreno, S. Moretto, A. Morreale, A. Morsch, V. Muccifora, E. Mudnic, D. Mühlheim, S. Muhuri, M. Mukherjee, J. D. Mulligan, M. G. Munhoz, K. Münning, R. H. Munzer, H. Murakami, S. Murray, L. Musa, J. Musinsky, C. J. Myers, B. Naik, R. Nair, B. K. Nandi, R. Nania, E. Nappi, M. U. Naru, H. Natal da Luz, C. Nattrass, S. R. Navarro, K. Nayak, R. Nayak, T. K. Nayak, S. Nazarenko, A. Nedosekin, R. A. Negrao De Oliveira, L. Nellen, S. V. Nesbo, F. Ng, M. Nicassio, M. Niculescu, J. Niedziela, B. S. Nielsen, S. Nikolaev, S. Nikulin, V. Nikulin, F. Noferini, P. Nomokonov, G. Nooren, J. C. C. Noris, J. Norman, A. Nyanin, J. Nystrand, H. Oeschler, S. Oh, A. Ohlson, T. Okubo, L. Olah, J. Oleniacz, A. C. Oliveira Da Silva, M. H. Oliver, J. Onderwaater, C. Oppedisano, R. Orava, M. Oravec, A. Ortiz Velasquez, A. Oskarsson, J. Otwinowski, K. Oyama, Y. Pachmayer, V. Pacik, D. Pagano, P. Pagano, G. Paić, P. Palni, J. Pan, A. K. Pandey, S. Panebianco, V. Papikyan, G. S. Pappalardo, P. Pareek, J. Park, W. J. Park, S. Parmar, A. Passfeld, S. P. Pathak, V. Paticchio, R. N. Patra, B. Paul, H. Pei, T. Peitzmann, X. Peng, L. G. Pereira, H. Pereira Da Costa, D. Peresunko, E. Perez Lezama, V. Peskov, Y. Pestov, V. Petráček, V. Petrov, M. Petrovici, C. Petta, R. P. Pezzi, S. Piano, M. Pikna, P. Pillot, L. O. D. L. Pimentel, O. Pinazza, L. Pinsky, D. B. Piyarathna, M. Płoskoń, M. Planinic, J. Pluta, S. Pochybova, P. L. M. Podesta-Lerma, M. G. Poghosyan, B. Polichtchouk, N. Poljak, W. Poonsawat, A. Pop, H. Poppenborg, S. Porteboeuf-Houssais, J. Porter, J. Pospisil, V. Pozdniakov, S. K. Prasad, R. Preghenella, F. Prino, C. A. Pruneau, I. Pshenichnov, M. Puccio, G. Puddu, P. Pujahari, V. Punin, J. Putschke, H. Qvigstad, A. Rachevski, S. Raha, S. Rajput, J. Rak, A. Rakotozafindrabe, L. Ramello, F. Rami, D. B. Rana, R. Raniwala, S. Raniwala, S. S. Räsänen, B. T. Rascanu, D. Rathee, V. Ratza, I. Ravasenga, K. F. Read, K. Redlich, A. Rehman, P. Reichelt, F. Reidt, X. Ren, R. Renfordt, A. R. Reolon, A. Reshetin, K. Reygers, V. Riabov, R. A. Ricci, T. Richert, M. Richter, P. Riedler, W. Riegler, F. Riggi, C. Ristea, M. Rodríguez Cahuantzi, K. Røed, E. Rogochaya, D. Rohr, D. Röhrich, P. S. Rokita, F. Ronchetti, L. Ronflette, P. Rosnet, A. Rossi, A. Rotondi, F. Roukoutakis, A. Roy, C. Roy, P. Roy, A. J. Rubio Montero, O. V. Rueda, R. Rui, R. Russo, A. Rustamov, E. Ryabinkin, Y. Ryabov, A. Rybicki, S. Saarinen, S. Sadhu, S. Sadovsky, K. Šafařík, S. K. Saha, B. Sahlmuller, B. Sahoo, P. Sahoo, R. Sahoo, S. Sahoo, P. K. Sahu, J. Saini, S. Sakai, M. A. Saleh, J. Salzwedel, S. Sambyal, V. Samsonov, A. Sandoval, D. Sarkar, N. Sarkar, P. Sarma, M. H. P. Sas, E. Scapparone, F. Scarlassara, R. P. Scharenberg, H. S. Scheid, C. Schiaua, R. Schicker, C. Schmidt, H. R. Schmidt, M. O. Schmidt, M. Schmidt, S. Schuchmann, J. Schukraft, Y. Schutz, K. Schwarz, K. Schweda, G. Scioli, E. Scomparin, R. Scott, M. Šefčík, J. E. Seger, Y. Sekiguchi, D. Sekihata, I. Selyuzhenkov, K. Senosi, S. Senyukov, E. Serradilla, P. Sett, A. Sevcenco, A. Shabanov, A. Shabetai, O. Shadura, R. Shahoyan, A. Shangaraev, A. Sharma, A. Sharma, M. Sharma, M. Sharma, N. Sharma, A. I. Sheikh, K. Shigaki, Q. Shou, K. Shtejer, Y. Sibiriak, S. Siddhanta, K. M. Sielewicz, T. Siemiarczuk, D. Silvermyr, C. Silvestre, G. Simatovic, G. Simonetti, R. Singaraju, R. Singh, V. Singhal, T. Sinha, B. Sitar, M. Sitta, T. B. Skaali, M. Slupecki, N. Smirnov, R. J. M. Snellings, T. W. Snellman, J. Song, M. Song, F. Soramel, S. Sorensen, F. Sozzi, E. Spiriti, I. Sputowska, B. K. Srivastava, J. Stachel, I. Stan, P. Stankus, E. Stenlund, J. H. Stiller, D. Stocco, P. Strmen, A. A. P. Suaide, T. Sugitate, C. Suire, M. Suleymanov, M. Suljic, R. Sultanov, M. Šumbera, S. Sumowidagdo, K. Suzuki, S. Swain, A. Szabo, I. Szarka, A. Szczepankiewicz, M. Szymanski, U. Tabassam, J. Takahashi, G. J. Tambave, N. Tanaka, M. Tarhini, M. Tariq, M. G. Tarzila, A. Tauro, G. Tejeda Muñoz, A. Telesca, K. Terasaki, C. Terrevoli, B. Teyssier, D. Thakur, S. Thakur, D. Thomas, R. Tieulent, A. Tikhonov, A. R. Timmins, A. Toia, S. Tripathy, S. Trogolo, G. Trombetta, V. Trubnikov, W. H. Trzaska, B. A. Trzeciak, T. Tsuji, A. Tumkin, R. Turrisi, T. S. Tveter, K. Ullaland, E. N. Umaka, A. Uras, G. L. Usai, A. Utrobicic, M. Vala, J. Van Der Maarel, J. W. Van Hoorne, M. van Leeuwen, T. Vanat, P. Vande Vyvre, D. Varga, A. Vargas, M. Vargyas, R. Varma, M. Vasileiou, A. Vasiliev, A. Vauthier, O. Vázquez Doce, V. Vechernin, A. M. Veen, A. Velure, E. Vercellin, S. Vergara Limón, R. Vernet, R. Vértesi, L. Vickovic, S. Vigolo, J. Viinikainen, Z. Vilakazi, O. Villalobos Baillie, A. Villatoro Tello, A. Vinogradov, L. Vinogradov, T. Virgili, V. Vislavicius, A. Vodopyanov, M. A. Völkl, K. Voloshin, S. A. Voloshin, G. Volpe, B. von Haller, I. Vorobyev, D. Voscek, D. Vranic, J. Vrláková, B. Wagner, J. Wagner, H. Wang, M. Wang, D. Watanabe, Y. Watanabe, M. Weber, S. G. Weber, D. F. Weiser, J. P. Wessels, U. Westerhoff, A. M. Whitehead, J. Wiechula, J. Wikne, G. Wilk, J. Wilkinson, G. A. Willems, M. C. S. Williams, B. Windelband, W. E. Witt, S. Yalcin, P. Yang, S. Yano, Z. Yin, H. Yokoyama, I.-K. Yoo, J. H. Yoon, V. Yurchenko, V. Zaccolo, A. Zaman, C. Zampolli, H. J. C. Zanoli, N. Zardoshti, A. Zarochentsev, P. Závada, N. Zaviyalov, H. Zbroszczyk, M. Zhalov, H. Zhang, X. Zhang, Y. Zhang, C. Zhang, Z. Zhang, C. Zhao, N. Zhigareva, D. Zhou, Y. Zhou, Z. Zhou, H. Zhu, J. Zhu, X. Zhu, A. Zichichi, A. Zimmermann, M. B. Zimmermann, S. Zimmermann, G. Zinovjev, J. Zmeskal, ALCE Collaboration

**Affiliations:** 1 A.I. Alikhanyan National Science Laboratory (Yerevan Physics Institute) Foundation, Yerevan, Armenia; 2 Benemérita Universidad Autónoma de Puebla, Puebla, Mexico; 3 Bogolyubov Institute for Theoretical Physics, Kiev, Ukraine; 4 Department of Physics, Centre for Astroparticle Physics and Space Science (CAPSS), Bose Institute, Kolkata, India; 5 Budker Institute for Nuclear Physics, Novosibirsk, Russia; 6 California Polytechnic State University, San Luis Obispo, CA USA; 7 Central China Normal University, Wuhan, China; 8 Centre de Calcul de l’IN2P3, Villeurbanne, Lyon, France; 9 Centro de Aplicaciones Tecnológicas y Desarrollo Nuclear (CEADEN), Havana, Cuba; 10 Centro de Investigaciones Energéticas Medioambientales y Tecnológicas (CIEMAT), Madrid, Spain; 11 Centro de Investigación y de Estudios Avanzados (CINVESTAV), Mexico City, Mérida, Mexico; 12 Centro Fermi-Museo Storico della Fisica e Centro Studi e Ricerche “Enrico Fermi’, Rome, Italy; 13 Chicago State University, Chicago, IL USA; 14 China Institute of Atomic Energy, Beijing, China; 15 COMSATS Institute of Information Technology (CIIT), Islamabad, Pakistan; 16 Departamento de Física de Partículas and IGFAE, Universidad de Santiago de Compostela, Santiago de Compostela, Spain; 17 Department of Physics, Aligarh Muslim University, Aligarh, India; 18 Department of Physics, Ohio State University, Columbus, OH USA; 19 Department of Physics, Sejong University, Seoul, South Korea; 20 Department of Physics, University of Oslo, Oslo, Norway; 21 Department of Physics and Technology, University of Bergen, Bergen, Norway; 22 Dipartimento di Fisica dell’Università ‘La Sapienza’ and Sezione INFN, Rome, Italy; 23 Dipartimento di Fisica dell’Università and Sezione INFN, Cagliari, Italy; 24 Dipartimento di Fisica dell’Università and Sezione INFN, Trieste, Italy; 25 Dipartimento di Fisica dell’Università and Sezione INFN, Turin, Italy; 26 Dipartimento di Fisica e Astronomia dell’Università and Sezione INFN, Bologna, Italy; 27 Dipartimento di Fisica e Astronomia dell’Università and Sezione INFN, Catania, Italy; 28 Dipartimento di Fisica e Astronomia dell’Università and Sezione INFN, Padua, Italy; 29 Dipartimento di Fisica ‘E.R. Caianiello’ dell’Università and Gruppo Collegato INFN, Salerno, Italy; 30 Dipartimento DISAT del Politecnico and Sezione INFN, Turin, Italy; 31 Dipartimento di Scienze e Innovazione Tecnologica dell’Università del Piemonte Orientale and INFN Sezione di Torino, Alessandria, Italy; 32 Dipartimento Interateneo di Fisica ‘M. Merlin’ and Sezione INFN, Bari, Italy; 33 Division of Experimental High Energy Physics, University of Lund, Lund, Sweden; 34 European Organization for Nuclear Research (CERN), Geneva, Switzerland; 35 Excellence Cluster Universe, Technische Universität München, Munich, Germany; 36 Faculty of Engineering, Bergen University College, Bergen, Norway; 37 Faculty of Mathematics, Physics and Informatics, Comenius University, Bratislava, Slovakia; 38 Faculty of Nuclear Sciences and Physical Engineering, Czech Technical University in Prague, Prague, Czech Republic; 39 Faculty of Science, P.J. Šafárik University, Kosice, Slovakia; 40 Faculty of Technology, Buskerud and Vestfold University College, Tonsberg, Norway; 41 Frankfurt Institute for Advanced Studies, Johann Wolfgang Goethe-Universität Frankfurt, Frankfurt, Germany; 42 Gangneung-Wonju National University, Gangneung, South Korea; 43 Department of Physics, Gauhati University, Guwahati, India; 44 Helmholtz-Institut für Strahlen- und Kernphysik, Rheinische Friedrich-Wilhelms-Universität Bonn, Bonn, Germany; 45 Helsinki Institute of Physics (HIP), Helsinki, Finland; 46 Hiroshima University, Hiroshima, Japan; 47 Indian Institute of Technology Bombay (IIT), Mumbai, India; 48 Indian Institute of Technology Indore, Indore, India; 49 Indonesian Institute of Sciences, Jakarta, Indonesia; 50 Inha University, Incheon, South Korea; 51 Institut de Physique Nucléaire d’Orsay (IPNO), Université Paris-Sud, CNRS-IN2P3, Orsay, France; 52 Institute for Nuclear Research, Academy of Sciences, Moscow, Russia; 53 Institute for Subatomic Physics of Utrecht University, Utrecht, The Netherlands; 54 Institute for Theoretical and Experimental Physics, Moscow, Russia; 55 Institute of Experimental Physics, Slovak Academy of Sciences, Kosice, Slovakia; 56 Institute of Physics, Academy of Sciences of the Czech Republic, Prague, Czech Republic; 57 Institute of Physics, Bhubaneswar, India; 58 Institute of Space Science (ISS), Bucharest, Romania; 59 Institut für Informatik, Johann Wolfgang Goethe-Universität Frankfurt, Frankfurt, Germany; 60 Institut für Kernphysik, Johann Wolfgang Goethe-Universität Frankfurt, Frankfurt, Germany; 61 Institut für Kernphysik, Westfälische Wilhelms-Universität Münster, Münster, Germany; 62 Instituto de Ciencias Nucleares, Universidad Nacional Autónoma de México, Mexico City, Mexico; 63 Instituto de Física, Universidade Federal do Rio Grande do Sul (UFRGS), Porto Alegre, Brazil; 64 Instituto de Física, Universidad Nacional Autónoma de México, Mexico City, Mexico; 65 IRFU, CEA, Université Paris-Saclay, 91191 Gif-sur-Yvette, Saclay, France; 66 iThemba LABS, National Research Foundation, Somerset West, South Africa; 67 Joint Institute for Nuclear Research (JINR), Dubna, Russia; 68 Konkuk University, Seoul, South Korea; 69 Korea Institute of Science and Technology Information, Daejeon, South Korea; 70 KTO Karatay University, Konya, Turkey; 71 Laboratoire de Physique Corpusculaire (LPC), Clermont Université, Université Blaise Pascal, CNRS-IN2P3, Clermont-Ferrand, France; 72 Laboratoire de Physique Subatomique et de Cosmologie, Université Grenoble-Alpes, CNRS-IN2P3, Grenoble, France; 73 Laboratori Nazionali di Frascati, INFN, Frascati, Italy; 74 Laboratori Nazionali di Legnaro, INFN, Legnaro, Italy; 75 Lawrence Berkeley National Laboratory, Berkeley, CA USA; 76 Moscow Engineering Physics Institute, Moscow, Russia; 77 Nagasaki Institute of Applied Science, Nagasaki, Japan; 78 Physics Department, National and Kapodistrian University of Athens, Athens, Greece; 79 National Centre for Nuclear Studies, Warsaw, Poland; 80 National Institute for Physics and Nuclear Engineering, Bucharest, Romania; 81 National Institute of Science Education and Research, Bhubaneswar, India; 82 National Nuclear Research Center, Baku, Azerbaijan; 83 National Research Centre Kurchatov Institute, Moscow, Russia; 84 Niels Bohr Institute, University of Copenhagen, Copenhagen, Denmark; 85 Nikhef, Nationaal instituut voor subatomaire fysica, Amsterdam, The Netherlands; 86 Nuclear Physics Group, STFC Daresbury Laboratory, Daresbury, UK; 87 Nuclear Physics Institute, Academy of Sciences of the Czech Republic, Řež u Prahy, Czech Republic; 88 Oak Ridge National Laboratory, Oak Ridge, TN USA; 89 Petersburg Nuclear Physics Institute, Gatchina, Russia; 90 Physics Department, Creighton University, Omaha, NE USA; 91 Physics Department, Panjab University, Chandigarh, India; 92 Physics Department, University of Cape Town, Cape Town, South Africa; 93 Physics Department, University of Jammu, Jammu, India; 94 Physics Department, University of Rajasthan, Jaipur, India; 95 Physikalisches Institut, Eberhard Karls Universität Tübingen, Tübingen, Germany; 96 Physikalisches Institut, Ruprecht-Karls-Universität Heidelberg, Heidelberg, Germany; 97 Physik Department, Technische Universität München, Munich, Germany; 98 Purdue University, West Lafayette, IN USA; 99 Pusan National University, Pusan, South Korea; 100 Research Division and ExtreMe Matter Institute EMMI, GSI Helmholtzzentrum für Schwerionenforschung GmbH, Darmstadt, Germany; 101 Rudjer Bošković Institute, Zagreb, Croatia; 102 Russian Federal Nuclear Center (VNIIEF), Sarov, Russia; 103 Saha Institute of Nuclear Physics, Kolkata, India; 104 School of Physics and Astronomy, University of Birmingham, Birmingham, UK; 105 Sección Física, Departamento de Ciencias, Pontificia Universidad Católica del Perú, Lima, Peru; 106 Sezione INFN, Bari, Italy; 107 Sezione INFN, Bologna, Italy; 108 Sezione INFN, Cagliari, Italy; 109 Sezione INFN, Catania, Italy; 110 Sezione INFN, Padua, Italy; 111 Sezione INFN, Rome, Italy; 112 Sezione INFN, Trieste, Italy; 113 Sezione INFN, Turin, Italy; 114 SSC IHEP of NRC Kurchatov Institute, Protvino, Russia; 115 Stefan Meyer Institut für Subatomare Physik (SMI), Vienna, Austria; 116 SUBATECH, IMT Atlantique, Université de Nantes, CNRS-IN2P3, Nantes, France; 117 Suranaree University of Technology, Nakhon Ratchasima, Thailand; 118 Technical University of Košice, Kosice, Slovakia; 119 Technical University of Split FESB, Split, Croatia; 120 The Henryk Niewodniczanski Institute of Nuclear Physics, Polish Academy of Sciences, Cracow, Poland; 121 Physics Department, The University of Texas at Austin, Austin, TX USA; 122 Universidad Autónoma de Sinaloa, Culiacán, Mexico; 123 Universidade de São Paulo (USP), São Paulo, Brazil; 124 Universidade Estadual de Campinas (UNICAMP), Campinas, Brazil; 125 Universidade Federal do ABC, Santo Andre, Brazil; 126 University of Houston, Houston, TX USA; 127 University of Jyväskylä, Jyväskylä, Finland; 128 University of Liverpool, Liverpool, UK; 129 University of Tennessee, Knoxville, TN USA; 130 University of the Witwatersrand, Johannesburg, South Africa; 131 University of Tokyo, Tokyo, Japan; 132 University of Tsukuba, Tsukuba, Japan; 133 University of Zagreb, Zagreb, Croatia; 134 Université de Lyon, Université Lyon 1, CNRS/IN2P3, IPN-Lyon, Villeurbanne, Lyon, France; 135 Université de Strasbourg, CNRS, IPHC UMR 7178, 67000 Strasbourg, France; 136 Università degli Studi di Pavia, Pavia, Italy; 137 Università di Brescia, Brescia, Italy; 138 V. Fock Institute for Physics, St. Petersburg State University, St. Petersburg, Russia; 139 Variable Energy Cyclotron Centre, Kolkata, India; 140 Warsaw University of Technology, Warsaw, Poland; 141 Wayne State University, Detroit, MI USA; 142 Wigner Research Centre for Physics, Hungarian Academy of Sciences, Budapest, Hungary; 143 Yale University, New Haven, CT USA; 144 Yonsei University, Seoul, South Korea; 145 Zentrum für Technologietransfer und Telekommunikation (ZTT), Fachhochschule Worms, Worms, Germany; 146 CERN, 1211 Geneva 23, Switzerland

## Abstract

We present results on transverse momentum ($p_{\mathrm {\textsc {t}}}$) and rapidity ($y$) differential production cross sections, mean transverse momentum and mean transverse momentum square of inclusive $\mathrm {J}/\psi$ and $\psi \mathrm {(2S)}$ at forward rapidity ($2.5<y<4$) as well as $\psi \mathrm {(2S)}$-to-$\mathrm {J}/\psi$ cross section ratios. These quantities are measured in pp collisions at center of mass energies $\sqrt{s}\,=5.02$ and 13 TeV with the ALICE detector. Both charmonium states are reconstructed in the dimuon decay channel, using the muon spectrometer. A comprehensive comparison to inclusive charmonium cross sections measured at $\sqrt{s}\,=2.76$, 7 and 8 TeV is performed. A comparison to non-relativistic quantum chromodynamics and fixed-order next-to-leading logarithm calculations, which describe prompt and non-prompt charmonium production respectively, is also presented. A good description of the data is obtained over the full $p_{\mathrm {\textsc {t}}}$ range, provided that both contributions are summed. In particular, it is found that for $p_{\mathrm {\textsc {t}}}>15$ GeV/*c* the non-prompt contribution reaches up to 50% of the total charmonium yield.

## Introduction

Charmonia, such as $\mathrm {J}/\psi$ and $\psi \mathrm {(2S)}$, are bound states of a charm and anti-charm quark ($c\bar{c}$). At LHC energies, their hadronic production results mostly from the hard scattering of two gluons into a $c\bar{c}$ pair followed by the evolution of this pair into a charmonium state. Charmonium measurements in pp collisions are essential to the investigation of their production mechanisms. They also provide a baseline for proton-nucleus and nucleus-nucleus results which in turn are used to quantify the properties of the quark-gluon plasma [1, 2].

Mainly three theoretical approaches are used to describe the hadronic production of charmonium: the Color Evaporation Model (CEM) [3, 4], the Color Singlet Model (CSM) [5] and the Non-Relativistic Quantum Chromo-Dynamics model (NRQCD) [6]. These approaches differ mainly in the treatment of the evolution of the heavy-quark pair into a bound state. In the CEM, the production cross section of a given charmonium is proportional to the $c\bar{c}$ cross section, integrated between the mass of the charmonium and twice the mass of the lightest D meson, with the proportionality factor being independent of the charmonium transverse momentum $p_{\mathrm {\textsc {t}}}$, rapidity *y* and of the collision center of mass energy $\sqrt{s}\,$. In the CSM, perturbative QCD is used to describe the $c\bar{c}$ production with the same quantum numbers as the final-state meson. In particular, only color-singlet (CS) $c\bar{c}$ pairs are considered. Finally, in the NRQCD framework charmonium can be formed from a $c\bar{c}$ pair produced either in a CS or in a color-octet (CO) state. The color neutralization of the CO state is treated as a non-perturbative process. For a given order in $\alpha _s$, it is expanded in powers of the relative velocity between the two charm quarks and parametrized using universal Long Distance Matrix Elements (LDME) which are fitted to the data. The predictive power of NRQCD calculations is tested by fitting the LDME to a subset of the data and comparing cross sections calculated with these LDME to measurements performed at different energies. It is therefore crucial to confront these models to as many measurements as possible, over a wide range of $p_{\mathrm {\textsc {t}}}$, *y* and $\sqrt{s}\,$, and with as many different charmonium states as possible. The comparison can also be extended to observables other than cross sections, such as charmonium polarization [7–9].

In this paper we present results on the production cross sections of inclusive $\mathrm {J}/\psi$ and $\psi \mathrm {(2S)}$ at forward rapidity ($2.5<y<4$) measured in pp collisions at center of mass energies $\sqrt{s}\,=13$ and 5.02 TeV. For $\mathrm {J}/\psi$ at $\sqrt{s}\,=5.02$ TeV, the $p_{\mathrm {\textsc {t}}}$-differential cross sections have been published in [10] while the *y*-differential cross sections are presented here for the first time.

The $\mathrm {J}/\psi$ and $\psi \mathrm {(2S)}$ are measured in the dimuon decay channel. The inclusive differential cross sections are obtained as a function of $p_{\mathrm {\textsc {t}}}$ and $y$ over the ranges $0<p_{\mathrm {\textsc {t}}}<30$ GeV/*c* for $\mathrm {J}/\psi$ at $\sqrt{s}\,=13$ TeV, $0<p_{\mathrm {\textsc {t}}}<12$ GeV/*c* for $\mathrm {J}/\psi$ at $\sqrt{s}\,=5.02$ TeV and $0<p_{\mathrm {\textsc {t}}}<16$ GeV/*c* for $\psi \mathrm {(2S)}$ at $\sqrt{s}\,=13$ TeV. At $\sqrt{s}\,=5.02$ TeV only the $p_{\mathrm {\textsc {t}}}$-integrated $\psi \mathrm {(2S)}$ cross section is measured due to the limited integrated luminosity. The $\mathrm {J}/\psi$ result at $\sqrt{s}\,=13$ TeV extends significantly the $p_{\mathrm {\textsc {t}}}$ reach of measurements performed in a similar rapidity range by LHCb [11]. The $\mathrm {J}/\psi$ result at $\sqrt{s}\,=5.02$ TeV and the $\psi \mathrm {(2S)}$ results at both $\sqrt{s}\,$ are the first at this rapidity. The inclusive $\psi \mathrm {(2S)}$-to-$\mathrm {J}/\psi$ cross section ratios as a function of both $p_{\mathrm {\textsc {t}}}$ and *y* are also presented. These results are compared to similar measurements performed at $\sqrt{s}\,=2.76$ [12], 7 [13] and 8 TeV [14]. These comparisons allow studying the variations of quantities such as the mean transverse momentum $\langle p_{\mathrm {\textsc {t}}} \rangle$, mean transverse momentum square $\langle p_{\mathrm {\textsc {t}}}^{2} \rangle$ and the $p_{\mathrm {\textsc {t}}}$-integrated cross section as a function of $\sqrt{s}\,$. Put together, these measurements constitute a stringent test for models of charmonium production. In particular, an extensive comparison of the $\mathrm {J}/\psi$ and $\psi \mathrm {(2S)}$ cross sections at all available collision energies to the calculations from two NRQCD groups is presented towards the end of the paper (Sect. 4). In addition, the $p_{\mathrm {\textsc {t}}}$-integrated $\mathrm {J}/\psi$ cross section as a function of $\sqrt{s}\,$ is also compared to a CEM calculation. No comparison to the CSM is performed since complete calculations are not available at these energies beside the ones published in [13, 15].

All cross sections reported in this paper are inclusive and contain, on top of the direct production of the charmonium, a contribution from the decay of heavier charmonium states as well as contributions from the decay of long-lived beauty flavored hadrons (*b*-hadrons). The first two contributions (direct production and decay from heavier charmonium states) are commonly called prompt, whereas the contribution from *b*-hadron decays is called non-prompt because of the large mean proper decay length of these hadrons ($\sim$ 500 $\upmu$m).

The paper is organized as follows: the ALICE apparatus and the data samples used for this analysis are described in Sect. 2, the analysis procedure is discussed in Sect. 3 while the results are presented and compared to measurements at different $\sqrt{s}\,$ as well as to models in Sect. 4.

## Apparatus and data samples

The ALICE detector is described in detail in [16, 17]. In this section, we introduce the detector subsystems relevant to the present analysis: the muon spectrometer, the Silicon Pixel Detector (SPD), the V0 scintillator hodoscopes and the T0 Cherenkov detectors.

The muon spectrometer [18] allows the detection and characterization of muons in the pseudorapidity range $-4<\eta <-2.5$.^1^ It consists of a ten-interaction-lengths front absorber followed by a 3 T m dipole magnet coupled to a system of tracking (MCH) and triggering (MTR) devices. The front absorber is placed between 0.9 and 5 m from the Interaction Point (IP) and filters out hadrons and low-momentum muons emitted at forward rapidity. Tracking in the MCH is performed using five stations, each one consisting of two planes of cathode pad chambers positioned between 5.2 and 14.4 m from the IP. The MTR is positioned downstream of a 1.2 m thick iron wall which absorbs the remaining hadrons that escape the front absorber as well as low-momentum muons. It is composed of two stations equipped with two planes of resistive plate chambers each placed at 16.1 and 17.1 m from the IP. A conical absorber ($\theta <2^\circ$) protects the muon spectrometer against secondary particles produced mainly by large-$\eta$ primary particles interacting with the beam pipe throughout its full length. Finally, a rear absorber located downstream of the spectrometer protects the MTR from the background generated by beam-gas interactions.

The SPD is used to reconstruct the primary vertex of the collision. It is a cylindrically-shaped silicon pixel tracker and corresponds to the two innermost layers of the Inner Tracking System (ITS) [19]. These two layers surround the beam pipe at average radii of 3.9 and 7.6 cm and cover the pseudorapidity intervals $|\eta |<2$ and $|\eta |<1.4$, respectively.

The V0 hodoscopes [20] consist of two scintillator arrays positioned on each side of the IP at $z=-90$ and 340 cm and covering the $\eta$ range $-3.7<\eta <-1.7$ and $2.8<\eta <5.1$ respectively. They are used for online triggering and to reject beam-gas events by means of offline timing cuts together with the T0 detectors.

Finally, the T0 detectors [21] are used for the luminosity determination. They consist of two arrays of quartz Cherenkov counters placed on both sides of the IP covering the $\eta$ ranges $-3.3<\eta <-3$ and $4.6<\eta <4.9$.

The data used for this paper were collected in 2015. They correspond to pp collisions at $\sqrt{s}\,=13$ and 5.02 TeV. The data at $\sqrt{s}\,=13$ TeV are divided into several sub-periods corresponding to different beam conditions and leading to different pile-up rates. The pile-up rate, defined as the probability that one recorded event contains two or more collisions, reaches up to 25% in the muon spectrometer for beams with the highest luminosity. The data at $\sqrt{s}\,=5.02$ TeV were collected during the 5 days immediately after the $\sqrt{s}\,=13$ TeV campaign. During this period the pile-up rate was stable and below 2.5%.

Events used for this analysis were collected using a dimuon trigger which requires that two muons of opposite sign are detected in the MTR in coincidence with the detection of a signal in each side of the V0. In addition, the transverse momentum $p_{\mathrm {\textsc {t}}}^{\mathrm {trig}}$ of each muon, evaluated online, is required to pass a threshold of 0.5 GeV/*c* (1 GeV/*c*) for the data taking at $\sqrt{s}\,=5.02$ (13) TeV in order to reject soft muons from $\pi$ and K decays and to limit the trigger rate when the instantaneous luminosity is high. This threshold is defined as the $p_{\mathrm {\textsc {t}}}$ value for which the single muon trigger efficiency reaches 50% [22].

The data samples available after the event selection described above correspond to an integrated luminosity ${L_\mathrm {int}}=3.19\pm 0.11$ pb$^{-1}$ and ${L_\mathrm {int}}=106.3\pm 2.2$ nb$^{-1}$ for $\sqrt{s}\,=13$ TeV and $\sqrt{s}\,=5.02$ TeV respectively. These integrated luminosities are measured following the procedure described in [23] for the data at $\sqrt{s}\,=13$ TeV and in [24] for those at $\sqrt{s}\,=5.02$ TeV. The systematic uncertainty on these quantities contains contributions from the measurement of the T0 trigger cross section using the Van der Meer scan technique [25] and the stability of the T0 trigger during data taking. The quadratic sum of these contributions amounts to 3.4% at $\sqrt{s}\,=13$ TeV and 2.1% at $\sqrt{s}\,=5.02$ TeV.

## Analysis

The differential production cross section for a charmonium state $\psi$ in a given $p_{\mathrm {\textsc {t}}}$ and *y* interval is:

1 $$\begin{aligned} \frac{{d }^2\sigma _{\psi }}{{d }p_{\mathrm {\textsc {t}}}{d }y}= \frac{1}{\Delta p_{\mathrm {\textsc {t}}}\Delta y}\frac{1}{L_\mathrm{int}}\frac{N_{\psi }(p_{\mathrm {\textsc {t}}},y)}{{{\mathrm {BR}}}_{\psi \rightarrow \mu ^+\mu ^-}A\varepsilon (p_{\mathrm {\textsc {t}}},y)}, \end{aligned}$$

where ${\mathrm {BR}}_{\psi \rightarrow \mu ^+\mu ^-}$ is the branching ratio of the charmonium state $\psi$ into a pair of muons ($5.96\pm 0.03$% for $\mathrm {J}/\psi$ and $0.79\pm 0.09$% for $\psi \mathrm {(2S)}$ [26]), $\Delta p_{\mathrm {\textsc {t}}}$ and $\Delta y$ are the widths of the $p_{\mathrm {\textsc {t}}}$ and *y* interval under consideration, $N_{\psi }(p_{\mathrm {\textsc {t}}},y)$ is the number of charmonia measured in this interval, $A\varepsilon (p_{\mathrm {\textsc {t}}},y)$ are the corresponding acceptance and efficiency corrections and ${L_\mathrm {int}}$ is the integrated luminosity of the data sample. The large pile-up rates mentioned in Sect. 2 for the $\sqrt{s}\,=13$ TeV data sample are accounted for in the calculation of ${L_\mathrm {int}}$ [23].

### Track selection

The number of charmonia in a given $p_{\mathrm {\textsc {t}}}$ and *y* interval is obtained by forming pairs of opposite-sign muon tracks detected in the muon spectrometer and by calculating the invariant mass of these pairs, ${m_{\mu \mu }}$. The resulting distribution is then fitted with several functions that account for both the charmonium signal and the background.

The procedure used to reconstruct muon candidates in the muon spectrometer is described in [18]. Once muon candidates are reconstructed, additional offline criteria are applied in order to improve the quality of the dimuon sample and the signal-to-background (S/B) ratio.

Tracks reconstructed in the MCH are required to match a track in the MTR which satisfies the single muon trigger condition mentioned in Sect. 2. Each muon candidate is required to have a pseudorapidity in the interval $-4<\eta <-2.5$ in order to match the acceptance of the muon spectrometer. Finally, a cut on the transverse coordinate of the muon ($R_\mathrm{abs}$) measured at the end of the front absorber, $17.5<R_{abs}<89$ cm, ensures that muons emitted at small angles and passing through the high density section of the front absorber are rejected.

These selection criteria remove most of the background tracks consisting of hadrons escaping from or produced in the front absorber, low-$p_{\mathrm {\textsc {t}}}$ muons from $\pi$ and K decays, secondary muons produced in the front absorber and fake tracks. They improve the S/B ratio by up to 30% for the $\mathrm {J}/\psi$ and by a factor 2 for $\psi \mathrm {(2S)}$.

### Signal extraction

In each dimuon $p_{\mathrm {\textsc {t}}}$ and *y* interval, several fits to the invariant mass distribution are performed over different invariant mass ranges and using various fitting functions in order to obtain the number of $\mathrm {J}/\psi$ and $\psi \mathrm {(2S)}$ and to evaluate the corresponding systematic uncertainty. In all cases, the fit function consists of a background to which two signal functions are added, one for the $\mathrm {J}/\psi$ and one for the $\psi \mathrm {(2S)}$.

**Fig. 1:**
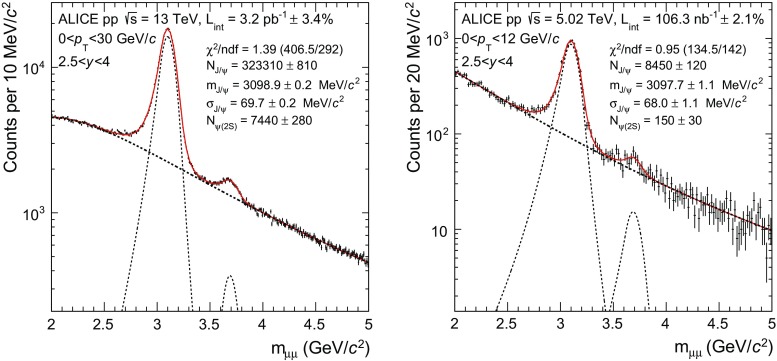
Example of fit to the opposite-sign dimuon invariant mass distributions in pp collisions at $\sqrt{s}\,=13$ TeV (*left*) and 5.02 TeV (*right*). *Dashed lines* correspond to either signal or background functions, whereas the *solid line* corresponds to the sum of the signal and background functions

At $\sqrt{s}\,=13$ TeV, the fits are performed over the invariant mass ranges $2.2<{m_{\mu \mu }}<4.5$ GeV/$c^2$ and $2<{m_{\mu \mu }}<5$ GeV/$c^2$. The background is described by either a pseudo-Gaussian function whose width varies linearly with the invariant mass or the product of a fourth-order polynomial and an exponential form. The $\mathrm {J}/\psi$ and $\psi \mathrm {(2S)}$ signals are described by the sum of either two Crystal Ball or two pseudo-Gaussian functions [27]. These two signal functions consist of a Gaussian core with tails added on the sides that fall off slower than a Gaussian function. In most $p_{\mathrm {\textsc {t}}}$ and *y* intervals the parameters entering the definition of these tails cannot be left free in the fit due to the poor S/B ratio in the corresponding invariant mass region. They are instead fixed either to the values obtained from Monte Carlo (MC) simulations described in Sect. 3.3, or to those obtained when fitting the measured $p_{\mathrm {\textsc {t}}}$- and *y*-integrated invariant mass distribution with these parameters left free. For the $\mathrm {J}/\psi$, the position, width and normalization of the signal are free parameters of the fit. For the $\psi \mathrm {(2S)}$ only the normalization is free, whereas the position and width are bound to those of the $\mathrm {J}/\psi$ following the same procedure as in [14]. Finally, in all fits the background parameters are left free.

An identical approach is used at $\sqrt{s}\,=5.02$ TeV, albeit with different invariant mass fitting ranges ($1.7<{m_{\mu \mu }}<4.8$ GeV/$c^2$ and $2<{m_{\mu \mu }}<4.4$ GeV/$c^2$) and a different set of background functions (a pseudo-Gaussian function or the ratio between a first- and a second-order polynomial function). For the signal the tails parameters are either fixed to those obtained in MC or taken from the $\sqrt{s}\,=13$ TeV analysis.

The number of charmonia measured in a given $p_{\mathrm {\textsc {t}}}$ and *y* interval and the corresponding statistical uncertainty are taken as the mean of the values and uncertainties obtained from all the fits performed in this interval. The root mean square of these values is used as a systematic uncertainty.

Examples of fits to the $p_{\mathrm {\textsc {t}}}$- and *y*-integrated invariant mass distributions are shown in Fig. 1, at $\sqrt{s}\,=13$ (left) and 5.02 TeV (right). About $331\times 10^3$ $\mathrm {J}/\psi$ and $8.1\times 10^3$ $\psi \mathrm {(2S)}$ are measured at $\sqrt{s}\,=13$ TeV whereas about $8.6\times 10^3$ $\mathrm {J}/\psi$ and 160 $\psi \mathrm {(2S)}$ are measured at $\sqrt{s}\,=5.02$ TeV. Corresponding S/B ratios, evaluated within three standard deviations with respect to the charmonium pole mass, are 3.4 (4.5) for $\mathrm {J}/\psi$ and 0.15 (0.18) for $\psi \mathrm {(2S)}$ at $\sqrt{s}\,=13$ (5.02) TeV.

### Acceptance and efficiency corrections

Acceptance and efficiency corrections are obtained using MC simulations by computing the ratio between the number of charmonia reconstructed in the muon spectrometer and the number of generated charmonia in the same $p_{\mathrm {\textsc {t}}}$ and *y* interval. Independent simulations are performed for $\mathrm {J}/\psi$ and $\psi \mathrm {(2S)}$ and for each collision energy. Charmonia are generated using input $p_{\mathrm {\textsc {t}}}$ and *y* distributions obtained iteratively from the data. They are decayed into two muons using EVTGEN [28] and PHOTOS [29] to properly account for the possible emission of accompanying radiative photons. It is assumed that both $\mathrm {J}/\psi$ and $\psi \mathrm {(2S)}$ are unpolarized consistently with the small longitudinal values reported in [7–9] and accounting for further dilution coming from non-prompt charmonia. The decay muons are tracked through a GEANT3 [30] model of the apparatus that includes a realistic description of the detectors and their performance during data taking. Track reconstruction and signal extraction are performed from the simulated hits generated in the detector using the same procedure and selection criteria as those used for the data.

The systematic uncertainty on acceptance and efficiency corrections contains the following contributions: (i) the parametrization of the input $p_{\mathrm {\textsc {t}}}$ and $y$ distributions, (ii) the uncertainty on the tracking efficiency in the MCH, (iii) the uncertainty on the MTR efficiency and (iv) the matching between tracks reconstructed in the MCH and tracks in the MTR.

For the parametrization of the MC input distributions, two sources of systematic uncertainty are considered: the correlations between $p_{\mathrm {\textsc {t}}}$ and *y* (more explicitly, the fact that the $p_{\mathrm {\textsc {t}}}$ distribution of a given charmonium state varies with the rapidity interval in which it is measured [11]) and the effect of finite statistics in the data used to parametrize these distributions. At $\sqrt{s}\,=5.02~$TeV, both contributions are evaluated by varying the input $p_{\mathrm {\textsc {t}}}$ and *y* distributions within limits that correspond to these effects and re-calculating the $A\varepsilon$ corrections in each case as done in [13]. This corresponds to a variation of the input yields of at most 15% as a function of *y* and 50% as a function of $p_{\mathrm {\textsc {t}}}$. For $\mathrm {J}/\psi$ measurements at $\sqrt{s}\,=13$ TeV a slightly different approach is adopted in order to further reduce the sensitivity of the simulations to the input $p_{\mathrm {\textsc {t}}}$ and *y* distributions. It consists in evaluating the acceptance and efficiency corrections in small 2-dimensional bins of *y* and $p_{\mathrm {\textsc {t}}}$. These corrections are then applied on a dimuon pair-by-pair basis when forming the invariant mass distribution rather than applying them on the total number of measured charmonia in a given (larger) $p_{\mathrm {\textsc {t}}}$ and *y* interval. For each pair the corrections that match its $p_{\mathrm {\textsc {t}}}$ and *y* are used, thus making the resulting $A\varepsilon$-corrected invariant mass distribution largely independent from the $p_{\mathrm {\textsc {t}}}$ and *y* distributions used as input to the simulations. For $\psi \mathrm {(2S)}$ this improved procedure is not applied because the uncertainties on the measurement are dominated by statistics and the same method as for $\mathrm {J}/\psi$ at $\sqrt{s}\,=5.02$ TeV is used instead.

The other three sources of systematic uncertainty (tracking efficiency in the MCH, MTR efficiency, and matching between MTR and MCH tracks) are evaluated using the same procedure as in [13], by comparing data and MC at the single muon level and propagating the observed differences to the dimuon case.

### Summary of the systematic uncertainties

Table 1 gives a summary of the relative systematic uncertainties on the charmonium cross sections measured at $\sqrt{s}\,=13$ and $\sqrt{s}\,=5.02$ TeV. The total systematic uncertainty is the quadratic sum of all the sources listed in this table. The uncertainty on the branching ratio is fully correlated between all measurements of a given state. The uncertainty on the integrated luminosity is fully correlated between measurements performed at the same $\sqrt{s}\,$ and considered as uncorrelated from one $\sqrt{s}\,$ to the other. The uncertainty on the signal extraction is considered as uncorrelated as a function of $p_{\mathrm {\textsc {t}}}$, *y* and $\sqrt{s}\,$, but partially correlated between $\mathrm {J}/\psi$ and $\psi \mathrm {(2S)}$. Finally, all other sources of uncertainty are considered as partially correlated across measurements at the same energy and uncorrelated from one energy to the other.

The systematic uncertainties on the MTR and MCH efficiencies are significantly smaller for the data at $\sqrt{s}\,=5.02$ TeV than at $\sqrt{s}\,=13$ TeV. This is due to the fact that the corresponding data taking period being very short, the detector conditions were more stable and therefore simpler to describe in the simulation.

**Table 1:** Relative systematic uncertainties associated to the $\mathrm {J}/\psi$ and $\psi \mathrm {(2S)}$ cross section measurements at $\sqrt{s}\,=13$ and 5.02 TeV. Values in parenthesis correspond to the minimum and maximum values as a function of $p_{\mathrm {\textsc {t}}}$ and *y*. For $\psi \mathrm {(2S)}$ at $\sqrt{s}\,=5.02$ TeV, only the $p_{\mathrm {\textsc {t}}}$-integrated values are reported

Source	$\sqrt{s}\,=13$ TeV		$\sqrt{s}\,=5.02$ TeV	
	$\mathrm {J}/\psi$ (%)	$\psi \mathrm {(2S)}$ (%)	$\mathrm {J}/\psi$ (%)	$\psi \mathrm {(2S)}$ (%)
Branching ratio	0.6	11	0.6	11
Luminosity	3.4	3.4	2.1	2.1
Signal extraction	3 (3–8)	5 (5–9)	3 (1.5–10)	8
MC input	0.5 (0.5–1.5)	1 (0.5–4)	2 (0.5–2.5)	2.5
MCH efficiency	4	4	1	1
MTR efficiency	4 (1.5–4)	4 (1.5–4)	2 (1.5–2)	2
Matching	1	1	1	1

## Results

### Cross sections and cross section ratios at $\sqrt{s}\,=~13$ and 5.02 TeV

**Fig. 2:**
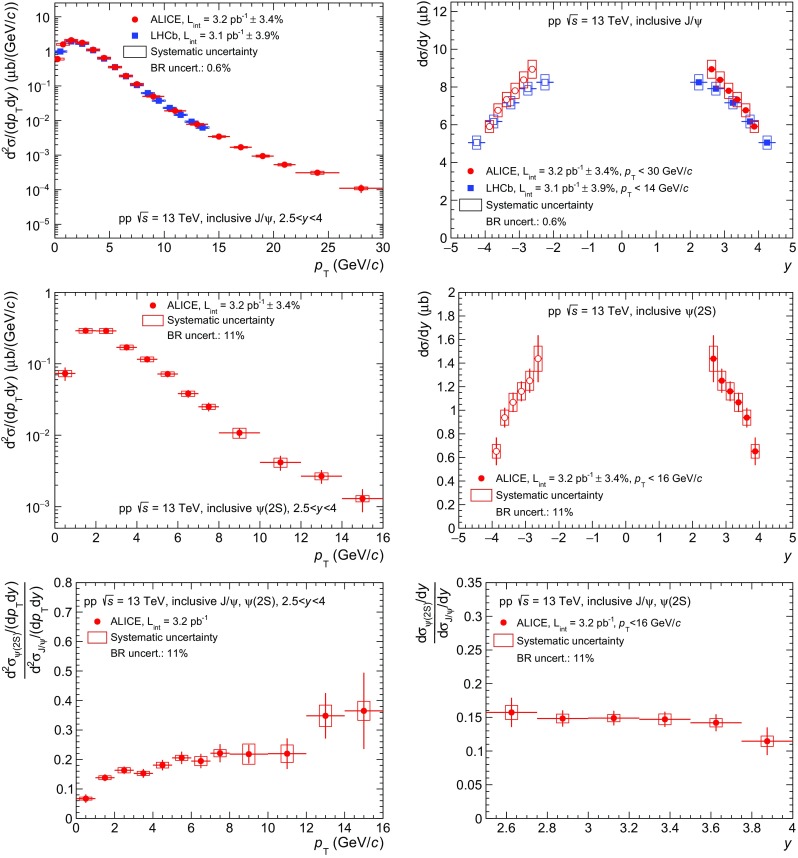
Inclusive $\mathrm {J}/\psi$ cross sections (*top*), $\psi \mathrm {(2S)}$ cross sections (*middle*) and $\psi \mathrm {(2S)}$-to-$\mathrm {J}/\psi$ cross section ratios (*bottom*) as a function of $p_{\mathrm {\textsc {t}}}$ (*left*) and $y$ (*right*) in pp collisions at $\sqrt{s}\,=13$ TeV. $\mathrm {J}/\psi$ cross sections are compared to LHCb measurements at the same $\sqrt{s}\,$ [11]. *Open symbols* are the reflection of the positive-*y* measurements with respect to $y=0$

Figure 2 summarizes the inclusive $\mathrm {J}/\psi$ and $\psi \mathrm {(2S)}$ cross sections measured by ALICE in pp collisions at $\sqrt{s}\,=13$ TeV as a function of the charmonium $p_{\mathrm {\textsc {t}}}$ (left column) and *y* (right column). The top row shows the $\mathrm {J}/\psi$ cross sections, middle row the $\psi \mathrm {(2S)}$ cross sections and bottom row the $\psi \mathrm {(2S)}$-to-$\mathrm {J}/\psi$ cross section ratios. In all figures except Figs. 5 and 6, systematic uncertainties are represented by boxes, while vertical lines are used for statistical uncertainties.

The $\mathrm {J}/\psi$ production cross sections as a function of $p_{\mathrm {\textsc {t}}}$ and $y$ are compared to measurements published by LHCb [11] at the same energy. The quoted LHCb values correspond to the sum of the prompt and the non-prompt contributions to the $\mathrm {J}/\psi$ production. For the comparison as a function of $p_{\mathrm {\textsc {t}}}$, the provided double-differential ($p_{\mathrm {\textsc {t}}}$ and *y*) cross sections are summed to match ALICE *y* coverage. The measurements of the two experiments are consistent within $1\sigma$ of their uncertainties. The ALICE measurement extends the $p_{\mathrm {\textsc {t}}}$ reach from 14 GeV/*c* to 30 GeV/*c* with respect to the LHCb results. For the $\psi \mathrm {(2S)}$ measurement, no comparisons are performed as this is the only measurement available to date at this energy and *y* range.

Systematic uncertainties on the signal extraction are reduced when forming the $\psi \mathrm {(2S)}$-to-$\mathrm {J}/\psi$ cross section ratios shown in the bottom panels of Fig. 2 due to correlations between the numerator and the denominator. All other sources of systematic uncertainties cancel except for the uncertainties on the MC input $p_{\mathrm {\textsc {t}}}$ and $y$ parametrizations. Measured ratios show a steady increase as a function of $p_{\mathrm {\textsc {t}}}$ and little or no dependence on *y* within uncertainties. This is also the case at lower $\sqrt{s}\,$ as it will be discussed in the next section.

**Fig. 3:**
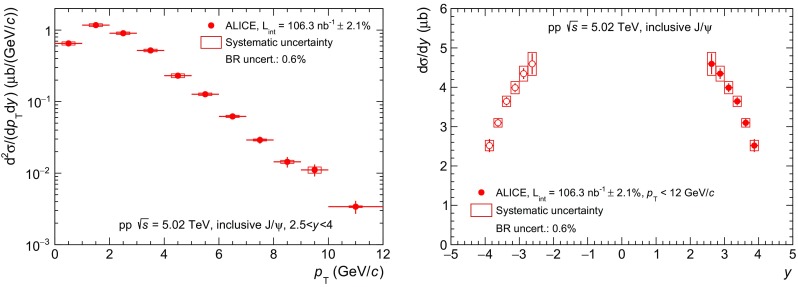
Inclusive $\mathrm {J}/\psi$ cross sections as function of $p_{\mathrm {\textsc {t}}}$ (*left*) and $y$ (*right*) in pp collisions at $\sqrt{s}\,=5.02$ TeV. *Open symbols* are the reflection of the positive-*y* measurements with respect to $y=0$

Figure 3 shows the inclusive $\mathrm {J}/\psi$ production cross section measurements performed by ALICE in pp collisions at $\sqrt{s}\,=5.02$ TeV as a function of $p_{\mathrm {\textsc {t}}}$ (left) and *y* (right). The $p_{\mathrm {\textsc {t}}}$-differential cross sections are published in [10] and serve as a reference for the $\mathrm {J}/\psi$ nuclear modification factors in $\mathrm {Pb\text{-- }Pb}$ collisions at the same $\sqrt{s}\,$. The *y*-differential cross sections are new to this analysis. Due to the limited integrated luminosity, only the $p_{\mathrm {\textsc {t}}}$- and *y*-integrated $\psi \mathrm {(2S)}$ cross section is measured using this data sample. It is discussed in the next section.

### Comparison to measurements at $\sqrt{s}\,=2.76$, 7 and 8 TeV

**Fig. 4:**
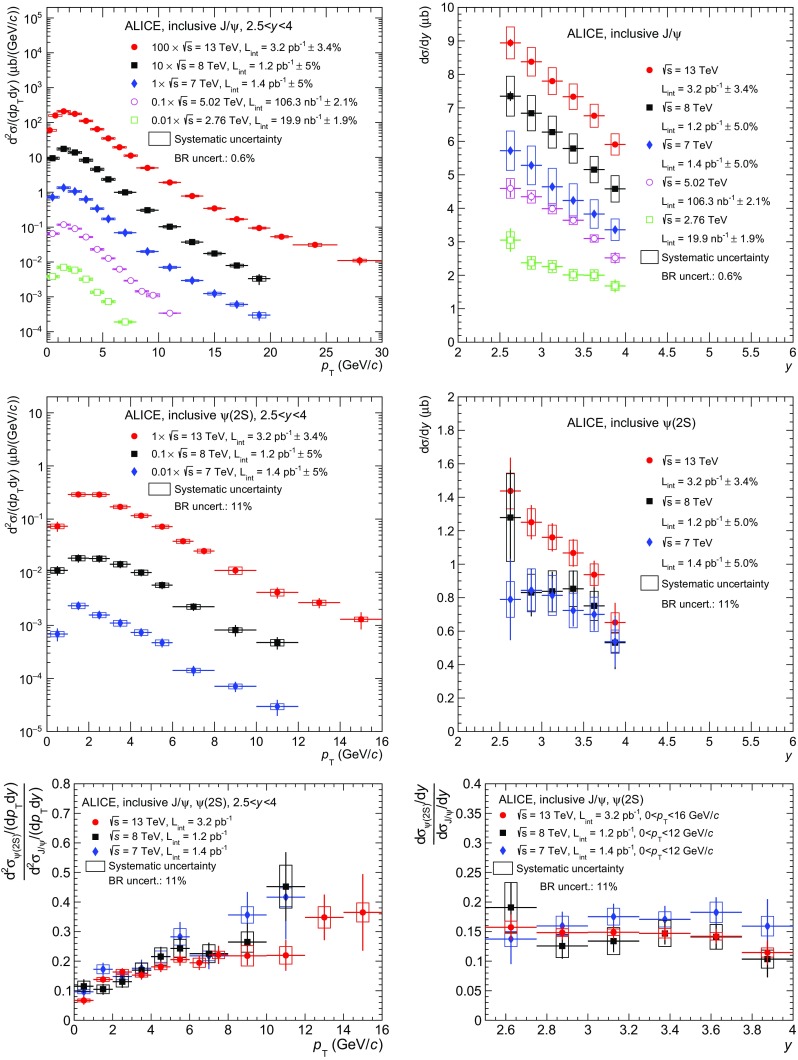
Inclusive $\mathrm {J}/\psi$ cross sections (*top*), $\psi \mathrm {(2S)}$ cross sections (*middle*) and $\psi \mathrm {(2S)}$-to-$\mathrm {J}/\psi$ cross section ratios (*bottom*) as function of $p_{\mathrm {\textsc {t}}}$ (*left*) and $y$ (*right*) in pp collisions at several values of $\sqrt{s}\,$

In Fig. 4, the cross sections and cross section ratios presented in the previous section are compared to other forward-*y* measurements in pp collisions at $\sqrt{s}\,=2.76$ [12], 7 [13] and 8 TeV [14]. We note that the integrated luminosity used for each measurement increases almost systematically with increasing $\sqrt{s}\,$, starting from 19.9 nb$^{-1}$ at $\sqrt{s}\,=2.76$ TeV up to 3.2 pb$^{-1}$ at $\sqrt{s}\,=13$ TeV. This, combined with the fact that the charmonium cross-section also increases with $\sqrt{s}$, has allowed to reach increasingly higher values of $p_{\mathrm {\textsc {t}}}$ for both $\mathrm {J}/\psi$ and $\psi \mathrm {(2S)}$ measurements. For the $\mathrm {J}/\psi$ this corresponds to an increase of the $p_{\mathrm {\textsc {t}}}$ reach from 8 GeV/*c* at $\sqrt{s}\,=2.76$ TeV up to 30 GeV/*c* at $\sqrt{s}\,=13$ TeV. For the $\psi \mathrm {(2S)}$ the corresponding increase goes from 12 GeV/*c* at $\sqrt{s}\,=7$ TeV to 16 GeV/*c* at $\sqrt{s}\,=13$ TeV.

The $\mathrm {J}/\psi$ $p_{\mathrm {\textsc {t}}}$-differential cross section measurements shown in the top-left panel of Fig. 4 indicate a hardening of the spectra with increasing $\sqrt{s}\,$. Also, for $\sqrt{s}\,\ge 7$ TeV, a change in the slope of the $p_{\mathrm {\textsc {t}}}$-differential cross section is visible for $p_{\mathrm {\textsc {t}}}> 10$ GeV/*c*. This change in slope is attributed to the onset of the contribution from non-prompt $\mathrm {J}/\psi$ to the inclusive cross section as it will be discussed in Sect. 4.3.

The corresponding $\psi \mathrm {(2S)}$ differential cross section measurements are shown in the middle panels of Fig. 4. The smaller cross sections with respect to $\mathrm {J}/\psi$ result in a smaller $p_{\mathrm {\textsc {t}}}$ reach as well as larger statistical uncertainties as a function of both $p_{\mathrm {\textsc {t}}}$ (left panel) and *y* (right panel).

In the bottom panels of Fig. 4 the measured $\psi \mathrm {(2S)}$-to-$\mathrm {J}/\psi$ cross section ratios are compared as a function of $p_{\mathrm {\textsc {t}}}$ (left) and *y* (right) for pp collisions at $\sqrt{s}=7$, 8 and 13 TeV. No significant change neither in shape nor magnitude of the ratio is observed among the three energies within the current uncertainties.

**Fig. 5:**
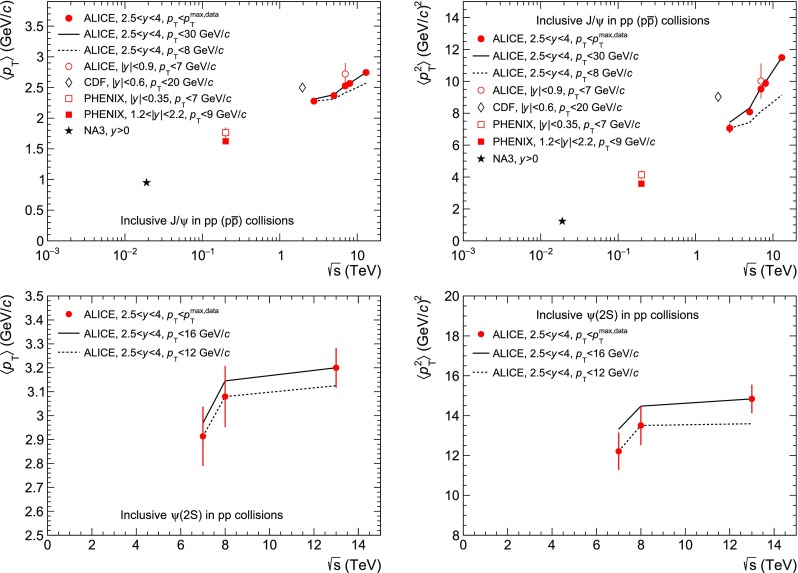
$\langle p_{\mathrm {\textsc {t}}} \rangle$ (*left*) and $\langle p_{\mathrm {\textsc {t}}}^{2} \rangle$ (*right*) as a function of $\sqrt{s}\,$ for $\mathrm {J}/\psi$ (*top*) and $\psi \mathrm {(2S)}$ (*bottom*). *Circles* correspond to ALICE data, while the *other symbols* correspond to measurements at lower $\sqrt{s}\,$. *Vertical lines* around the data points correspond to the quadratic sum of the statistical and uncorrelated systematic uncertainties. The *solid lines* correspond to calculating $\langle p_{\mathrm {\textsc {t}}} \rangle$ and $\langle p_{\mathrm {\textsc {t}}}^{2} \rangle$ when extrapolating the $p_{\mathrm {\textsc {t}}}$ coverage to the largest available range in ALICE data ($0<p_{\mathrm {\textsc {t}}}<30$ GeV/*c* for $\mathrm {J}/\psi$ and $0<p_{\mathrm {\textsc {t}}}<16$ GeV/*c* for $\psi \mathrm {(2S)}$), while the *dashed lines* correspond to truncating the data to the smallest $p_{\mathrm {\textsc {t}}}$ range available ($0<p_{\mathrm {\textsc {t}}}<8$ GeV/*c* for $\mathrm {J}/\psi$ and $0<p_{\mathrm {\textsc {t}}}<12$ GeV/*c* for $\psi \mathrm {(2S)}$)

To better quantify the hardening of the $\mathrm {J}/\psi$ and $\psi \mathrm {(2S)}$ $p_{\mathrm {\textsc {t}}}$ spectra with increasing $\sqrt{s}\,$, a computation of the corresponding mean transverse momentum $\langle p_{\mathrm {\textsc {t}}} \rangle$ and mean transverse momentum square $\langle p_{\mathrm {\textsc {t}}}^{2} \rangle$ is performed. This is achieved by fitting the $\mathrm {J}/\psi$ and $\psi \mathrm {(2S)}$ $p_{\mathrm {\textsc {t}}}$-differential cross sections with the following function:

2 $$\begin{aligned} f(p_{\mathrm {\textsc {t}}}) = C \times \frac{ p_{\mathrm {\textsc {t}}}}{ {\left( 1 + {\left( \frac{p_{\mathrm {\textsc {t}}}}{ p_0 } \right) }^2 \right) }^n }, \end{aligned}$$

with the parameters *C*, $p_0$ and *n* left free.

The $\langle p_{\mathrm {\textsc {t}}} \rangle$ and $\langle p_{\mathrm {\textsc {t}}}^{2} \rangle$ are then obtained as the first and second moments of the above function in a given $p_{\mathrm {\textsc {t}}}$ range. The uncertainty on these quantities is evaluated by multiplying the covariance matrix of the fit on each side by the relevant Jacobian matrix, evaluated numerically and taking the square root of the result. This is performed either considering separately the statistical and uncorrelated systematic uncertainties, or by using their quadratic sum in order to obtain the corresponding statistical, systematic or total uncertainty. A similar approach was adopted in [12].

**Fig. 6:**
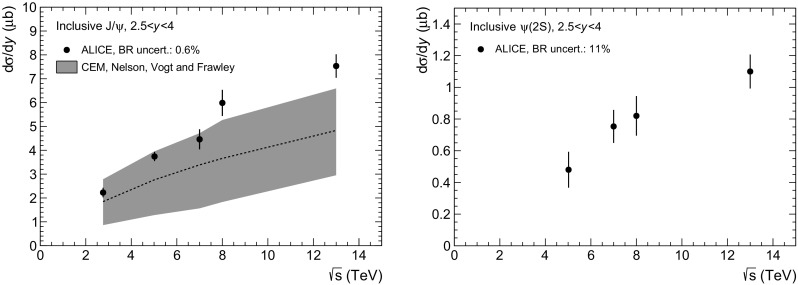
$\mathrm {J}/\psi$ (*left*) and $\psi \mathrm {(2S)}$ (*right*) inclusive cross section ${d }\sigma /{d }y$ as a function of $\sqrt{s}\,$. *Vertical lines* correspond to the quadratic sum of the statistical and uncorrelated systematic uncertainties. $\mathrm {J}/\psi$ cross sections are compared to a CEM calculation from [34]

Figure 5 shows the $\langle p_{\mathrm {\textsc {t}}} \rangle$ (left) and $\langle p_{\mathrm {\textsc {t}}}^{2} \rangle$ (right) results for $\mathrm {J}/\psi$ (top) and $\psi \mathrm {(2S)}$ (bottom). In this figure as well as in Fig. 6, the vertical lines correspond to the quadratic sum of the statistical and uncorrelated systematic uncertainties.

For $\mathrm {J}/\psi$ at $\sqrt{s}\,=2.76$ TeV the value from [12] is used. At $\sqrt{s}\,=7$ TeV the data from [13] are used instead of the result from [12] because the available integrated luminosity is much larger ($\times 90$) and the $p_{\mathrm {\textsc {t}}}$ reach increased from 8 to 20 GeV/*c*. It was checked that both results are consistent when truncated to the same $p_{\mathrm {\textsc {t}}}$ range. At $\sqrt{s}\,=8$ TeV the data from [14] are used, while for $\sqrt{s}\,=5.02$ and 13 TeV the results are from this analysis.

In the top panels of Fig. 5, ALICE measurements are compared to lower energy results from CDF [31], PHENIX [32] and NA3 [33]. A steady increase of $\langle p_{\mathrm {\textsc {t}}} \rangle$ and $\langle p_{\mathrm {\textsc {t}}}^{2} \rangle$ is observed with increasing $\sqrt{s}\,$. This is consistent with the expected hardening of the corresponding $p_{\mathrm {\textsc {t}}}$ distributions. Moreover, values at mid- are systematically larger than at forward-rapidity. As discussed in [32], this observation could be attributed to an increase in the longitudinal momentum at forward-rapidity leaving less energy available in the transverse plane. The bottom panels of Fig. 5 show the corresponding measurements for $\psi \mathrm {(2S)}$ at $\sqrt{s}\,=7$, 8 and 13 TeV. An increase with $\sqrt{s}\,$ is also observed similar to that of the $\mathrm {J}/\psi$.

Part of the increase observed for ALICE measurements shown in all four panels of Fig. 5 is due to the fact that the $p_{\mathrm {\textsc {t}}}$ range used for evaluating $\langle p_{\mathrm {\textsc {t}}} \rangle$ and $\langle p_{\mathrm {\textsc {t}}}^{2} \rangle$, chosen to be the same as in the corresponding data, also increases with $\sqrt{s}\,$. To illustrate this effect, these quantities were re-calculated either when truncating the data to the smallest available $p_{\mathrm {\textsc {t}}}$ range ($0<p_{\mathrm {\textsc {t}}}<8$ GeV/*c* for $\mathrm {J}/\psi$ and $0<p_{\mathrm {\textsc {t}}}<12$ GeV/*c* for $\psi \mathrm {(2S)}$) or when using the fit based on Eq. 2 to extrapolate the data to the largest available range ($0<p_{\mathrm {\textsc {t}}}<30$ GeV/*c* for $\mathrm {J}/\psi$ and $0<p_{\mathrm {\textsc {t}}}<16$ GeV/c for $\psi \mathrm {(2S)}$). The resulting values are shown in the figures as dashed lines for the truncation and solid lines for the extrapolation. In all cases the observed increasing trend still holds.

Finally, Fig. 6 shows the $\mathrm {J}/\psi$ (left) and $\psi \mathrm {(2S)}$ (right) $p_{\mathrm {\textsc {t}}}$- and *y*-integrated inclusive cross sections as a function of $\sqrt{s}\,$, measured by ALICE in the *y* range $2.5<y<4$. For both particles a steady increase of $\mathrm{d}\sigma /\mathrm{d}y$ is observed as a function of increasing $\sqrt{s}\,$. For the $\mathrm {J}/\psi$, the cross sections are compared to a calculation done by Nelson, Vogt and Frawley in the CEM framework [34]. While the data and the model are compatible within uncertainties, the data lie on the upper side of the calculation and the difference to the central value becomes larger with increasing $\sqrt{s}\,$.

### Comparisons to models

**Fig. 7:**
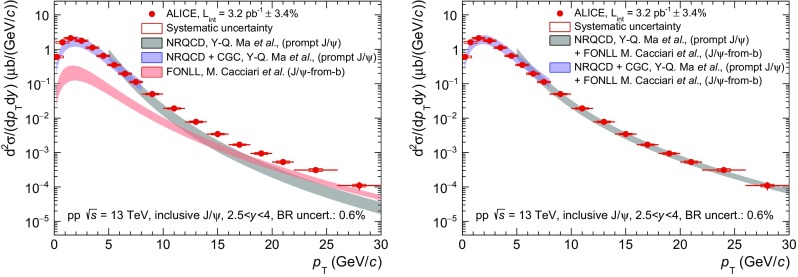
*Left panel* $\mathrm {J}/\psi$ differential cross sections (*red circles*) in pp collisions at $\sqrt{s}\,=13$ TeV compared to NLO NRQCD (*grey*) [35], LO NRQCD coupled with CGC (*blue*) [36] and FONLL (*red*) [37]. *Right panel* The non-prompt $\mathrm {J}/\psi$ contribution estimated with FONLL is summed to the two calculations for prompt $\mathrm {J}/\psi$ production and compared to the same data

As discussed in the introduction, all ALICE $\mathrm {J}/\psi$ and $\psi \mathrm {(2S)}$ measurements presented in this paper are inclusive and consist of a prompt and a non-prompt contribution. In order to compare model calculations to the data both contributions must be accounted for. This is illustrated in Fig. 7 for the $\mathrm {J}/\psi$ production cross section as a function of $p_{\mathrm {\textsc {t}}}$ in pp collisions at $\sqrt{s}\,=13$ TeV.

In the left panel of Fig. 7, ALICE data are compared to three calculations: (i) in grey to a prompt $\mathrm {J}/\psi$ Next-to-Leading-Order (NLO) NRQCD calculation from Ma, Wang and Chao [35], (ii) in blue to a prompt $\mathrm {J}/\psi$ Leading Order (LO) NRQCD calculation coupled to a Color Glass Condensate (CGC) description of the low-*x* gluons in the proton from Ma and Venugopalan [36] and (iii) in red to a non-prompt $\mathrm {J}/\psi$ Fixed-Order Next-to-Leading Logarithm (FONLL) calculation by Cacciari et al. [37].

Both NRQCD prompt $\mathrm {J}/\psi$ calculations account for the decay of $\psi \mathrm {(2S)}$ and $\chi _c$ into $\mathrm {J}/\psi$.

For $p_{\mathrm {\textsc {t}}}<8$ GeV/*c* where the contribution from non-prompt $\mathrm {J}/\psi$ estimated using FONLL is below 10%, the NRQCD+CGC prompt $\mathrm {J}/\psi$ calculation reproduces the data reasonably well. For higher $p_{\mathrm {\textsc {t}}}$ on the other hand, the NLO NRQCD calculation underestimates the measured cross sections and the disagreement increases with increasing $p_{\mathrm {\textsc {t}}}$. This disagreement is explained by the corresponding increase of the non-prompt $\mathrm {J}/\psi$ contribution, which according to FONLL, becomes as high as the prompt contribution and even exceeds it for $p_{\mathrm {\textsc {t}}}>15$ GeV/*c*. This is consistent with the measured non-prompt $\mathrm {J}/\psi$ fractions reported by LHCb in [11].

In the right panel of Fig. 7, the NRQCD and FONLL calculations for prompt and non-prompt $\mathrm {J}/\psi$ production are summed in order to obtain an ad hoc model of inclusive $\mathrm {J}/\psi$ production. The sum is performed separately for the NRQCD+CGC calculation at low $p_{\mathrm {\textsc {t}}}$ and the NLO NRQCD at high $p_{\mathrm {\textsc {t}}}$. In both cases, the uncertainties on FONLL and NRQCD are considered as uncorrelated when calculating the uncertainty band on the sum. This is motivated by the fact that the NRQCD calculations refer to the production of charm quarks and charmed mesons, while the FONLL calculation applies to the production of beauty quarks and *b*-hadrons which are then decayed into $\mathrm {J}/\psi$ mesons. A good description of the data is obtained over the full $p_{\mathrm {\textsc {t}}}$ range and spanning more than four orders of magnitude in the cross sections.

**Fig. 8:**
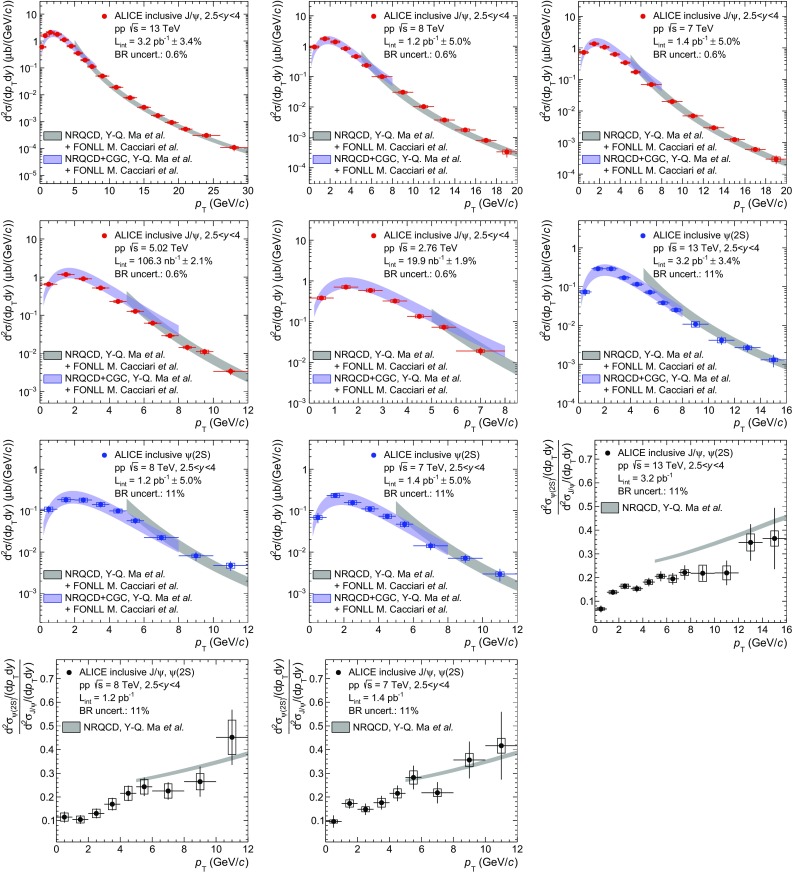
Comparisons between ALICE $\mathrm {J}/\psi$ and $\psi \mathrm {(2S)}$ data and summed NRQCD and FONLL model calculations from [35–37]. The first five panels correspond to inclusive $\mathrm {J}/\psi$ production cross sections as a function of $p_{\mathrm {\textsc {t}}}$ in pp collisions at $\sqrt{s}\,=13$, 8, 7, 5.02 and 2.76 TeV (*red*), the next three panels to inclusive $\psi \mathrm {(2S)}$ cross sections as a function of $p_{\mathrm {\textsc {t}}}$ at $\sqrt{s}\,=13$, 8 and 7 TeV (*blue*) and the last three panels to $\psi \mathrm {(2S)}$-to-$\mathrm {J}/\psi$ cross section ratios as a function of $p_{\mathrm {\textsc {t}}}$ at the same $\sqrt{s}\,$ (*black*)

The same groups have also provided NRQCD calculations for inclusive $\mathrm {J}/\psi$ production in pp collisions at $\sqrt{s}\,=8$, 7, 5.02 and 2.76 TeV, and for $\psi \mathrm {(2S)}$ at $\sqrt{s}\,=13$, 8 and 7 TeV. These calculations are compared to ALICE measurements in Fig. 8. Also shown in this figure are comparisons from the high-$p_{\mathrm {\textsc {t}}}$ NLO NRQCD calculations to ALICE $\psi \mathrm {(2S)}$-to-$\mathrm {J}/\psi$ cross section ratios as a function of $p_{\mathrm {\textsc {t}}}$. The motivation for showing this comparison of the cross section ratios is that many of the systematic uncertainties cancel both for the data (as discussed in Sect. 4.1) and for the theory.

Except for the cross section ratios, in all other panels the same strategy as in Fig. 7 is applied and the non-prompt contribution to inclusive charmonium production is added to the model using FONLL before comparing to the data. The FONLL+NRQCD summation is not performed for $\psi \mathrm {(2S)}$-to-$\mathrm {J}/\psi$ cross section ratios due to the added complexity introduced by the estimation of the error cancellation between the models. Moreover, the impact of the non-prompt charmonium contribution on these ratios is expected to be small because it enters both the numerator and the denominator with a similar magnitude (according to FONLL) and largely cancels out. We note that similar high-$p_{\mathrm {\textsc {t}}}$ NLO NRQCD calculations [38] were already compared to ALICE $\mathrm {J}/\psi$ and $\psi \mathrm {(2S)}$ cross sections at $\sqrt{s}\,=7$ TeV in [13], albeit with a different strategy to account for the non-prompt charmonia.

**Fig. 9:**
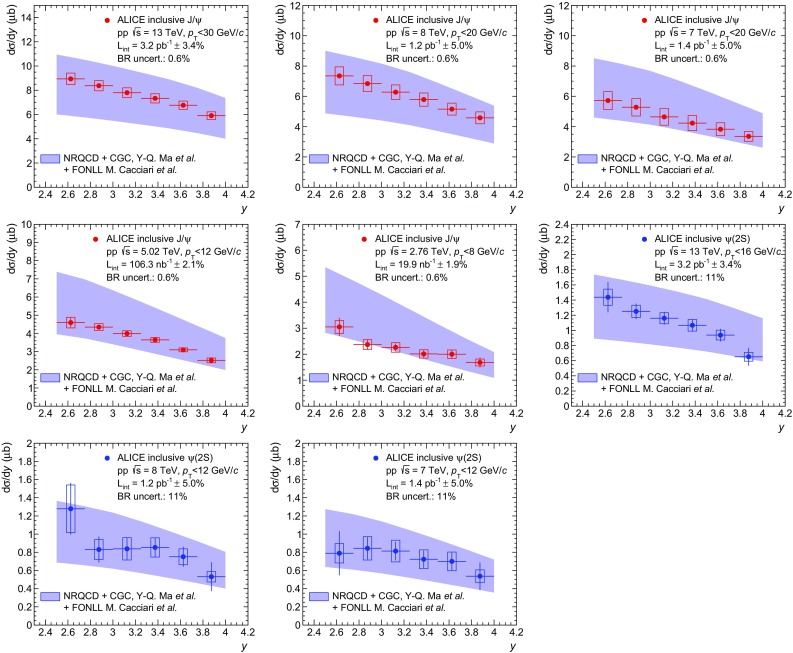
Comparisons between ALICE $\mathrm {J}/\psi$ and $\psi \mathrm {(2S)}$ data and summed NRQCD and FONLL model calculations from [36, 37]. The first five panels correspond to inclusive $\mathrm {J}/\psi$ production cross sections as a function of *y* in pp collisions at $\sqrt{s}\,=13$, 8 and 7, 5.02 and 2.76 TeV (*red*), while the next three panels to inclusive $\psi \mathrm {(2S)}$ cross sections as a function of *y* at $\sqrt{s}\,=13$, 8 and 7 TeV (*blue*)

Since the NRQCD+CGC calculation from [36] extends down to zero $p_{\mathrm {\textsc {t}}}$, it can be integrated over $p_{\mathrm {\textsc {t}}}$ and directly compared to ALICE $p_{\mathrm {\textsc {t}}}$-integrated cross sections as a function of *y*. This calculation neglects the contribution from charmonium with $p_{\mathrm {\textsc {t}}}>8$ GeV/*c* to the total cross section, which anyway contributes by less than 3%. The results of this comparison as a function of *y* are shown in Fig. 9.

Overall, a good agreement between the model and the data is observed for all measured cross sections, for both $\mathrm {J}/\psi$ and $\psi \mathrm {(2S)}$ as a function of either $p_{\mathrm {\textsc {t}}}$ or *y* and for all the collision energies considered. For $\psi \mathrm {(2S)}$-to-$\mathrm {J}/\psi$ cross section ratios as a function of $p_{\mathrm {\textsc {t}}}$ however, the model tends to be slightly above the data especially at $\sqrt{s}\,=13$ TeV. This tension appears mainly because of the error cancellation between the uncertainties on the $\mathrm {J}/\psi$ and $\psi \mathrm {(2S)}$ cross sections mentioned above.

**Fig. 10:**
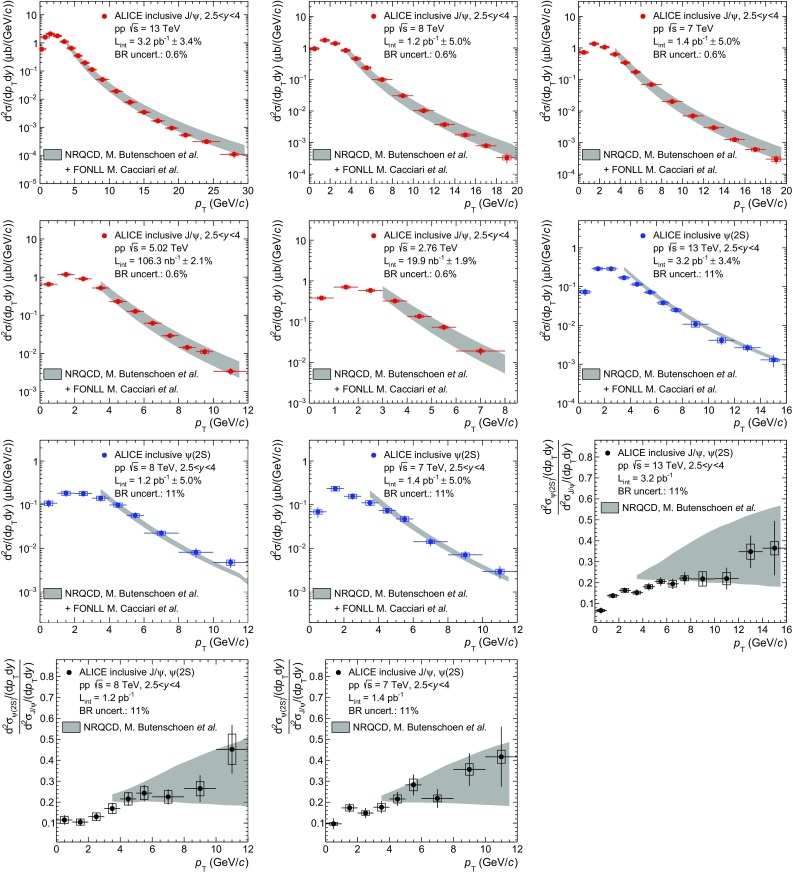
Comparisons between ALICE $\mathrm {J}/\psi$ and $\psi \mathrm {(2S)}$ data and summed NRQCD and FONLL model calculations from [37, 39]. The first five panels correspond to inclusive $\mathrm {J}/\psi$ production cross sections as a function of $p_{\mathrm {\textsc {t}}}$ in pp collisions at $\sqrt{s}\,=13$, 8, 7, 5.02 and 2.76 TeV (*red*), the next three panels to inclusive $\psi \mathrm {(2S)}$ cross sections as a function of $p_{\mathrm {\textsc {t}}}$ at $\sqrt{s}\,=13$, 8 and 7 TeV (*blue*) and the last three panels to $\psi \mathrm {(2S)}$-to-$\mathrm {J}/\psi$ cross section ratios as a function of $p_{\mathrm {\textsc {t}}}$ at the same $\sqrt{s}\,$ (*black*)

In Fig. 10, the ALICE measurements are compared to a second set of NLO NRQCD calculations from Butenschön and Kniehl [39]. In this case only high-$p_{\mathrm {\textsc {t}}}$ calculations ($p_{\mathrm {\textsc {t}}}>3$ GeV/*c*) are available. The ALICE $p_{\mathrm {\textsc {t}}}$-integrated cross sections as a function of *y* cannot be thus compared to the theory due to this $p_{\mathrm {\textsc {t}}}$ cut. As was the case for the comparisons shown in Figs. 8 and 9, FONLL is used to estimate the contribution from non-prompt charmonium production and added to the NRQCD calculation.

The two NLO NRQCD calculations from Butenschön and Kniehl (Fig. 10) and from Ma, Wang and Chao (Fig. 8) differ in the parametrization of the Long Distance Matrix Elements (LDME) used to calculate the color-octet contributions to the charmonium production cross section. The first calculation uses three matrix elements whereas the second uses only two linear combinations of these three elements. Other differences include: the data sets used to fit these matrix elements, the minimum $p_{\mathrm {\textsc {t}}}$ above which the calculation is applicable and the way by which contributions from $\chi _c$ and $\psi \mathrm {(2S)}$ decays to prompt $\mathrm {J}/\psi$ production are accounted for.

Although the agreement between the model and the data is of similar quality in Fig. 8 and 10, some differences are visible. In particular, in Fig. 10, the calculation tends to overestimate the measured $\mathrm {J}/\psi$ cross sections towards high-$p_{\mathrm {\textsc {t}}}$ and the uncertainties are larger than in Fig. 8. The uncertainties on the $\psi \mathrm {(2S)}$-to-$\mathrm {J}/\psi$ cross section ratios are also significantly larger and consequently the agreement to the data is better. These observations are a consequence of the differences between the two calculations detailed above and in particular the fact that the fits of the LDME start at a lower $p_{\mathrm {\textsc {t}}}$ and include a larger number of data sets in the second case.

## Conclusions

The inclusive $\mathrm {J}/\psi$ and $\psi \mathrm {(2S)}$ differential cross sections as well as $\psi \mathrm {(2S)}$-to-$\mathrm {J}/\psi$ cross section ratios as a function of $p_{\mathrm {\textsc {t}}}$ and *y* have been measured in pp collisions at $\sqrt{s}\,=5.02$ and 13 TeV with the ALICE detector. Combined with similar measurements performed at $\sqrt{s}\,=2.76$ [12], 7 [13] and 8 TeV [14], these results constitute a stringent test for models of charmonium production and allow the study of quantities such as $\langle p_{\mathrm {\textsc {t}}} \rangle$, $\langle p_{\mathrm {\textsc {t}}}^{2} \rangle$ and $p_{\mathrm {\textsc {t}}}$-integrated ${d }\sigma /{d }y$ as a function of $\sqrt{s}\,$.

The results at $\sqrt{s}\,=$13 TeV significantly extend the $p_{\mathrm {\textsc {t}}}$ reach for both charmonium states with respect to measurements performed by ALICE at lower energies, up to 30 GeV/*c* for the $\mathrm {J}/\psi$ and 16 GeV/*c* for the $\psi \mathrm {(2S)}$. When comparing the $\mathrm {J}/\psi$ cross sections vs $p_{\mathrm {\textsc {t}}}$ to measurements at lower $\sqrt{s}\,$, a hardening of the spectra is observed with increasing collision energy. This is confirmed by measurements of the $\mathrm {J}/\psi$ $\langle p_{\mathrm {\textsc {t}}} \rangle$ and $\langle p_{\mathrm {\textsc {t}}}^{2} \rangle$, while a similar trend is observed for the $\psi \mathrm {(2S)}$. Regarding inclusive $\psi \mathrm {(2S)}$-to-$\mathrm {J}/\psi$ cross section ratios, no $\sqrt{s}\,$ dependence is observed within uncertainties.

Comparisons of $\mathrm {J}/\psi$ and $\psi \mathrm {(2S)}$ cross sections and cross section ratios as a function of both $p_{\mathrm {\textsc {t}}}$ and *y* to NLO NRQCD and LO NRQCD+CGC prompt-charmonium calculations have been presented for all available collision energies. Concerning the $\mathrm {J}/\psi$ cross section as a function of $p_{\mathrm {\textsc {t}}}$, an excellent agreement is observed between data and theory, provided that the non-prompt contribution to the inclusive cross section is included using FONLL. This comparison indicates that for $p_{\mathrm {\textsc {t}}}>15$ GeV/*c*, the non-prompt contribution can reach up to 50%. An overall good agreement is also observed for $\psi \mathrm {(2S)}$ production and for the cross sections as a function of $y$ albeit with larger uncertainties.

With the large contribution from non-prompt $\mathrm {J}/\psi$ to the inclusive cross sections observed for high $p_{\mathrm {\textsc {t}}}$ at $\sqrt{s}=13$ TeV, it is of relatively little interest to try to further extend the $p_{\mathrm {\textsc {t}}}$ reach of the inclusive measurement for understanding charmonium production. This is as long as one is not capable of separating experimentally the prompt and the non-prompt contributions and relies on models instead. This separation will become possible in ALICE starting from 2021 with the addition of the Muon Forward Tracker [40].

## Footnotes

1 We note that the ALICE reference frame defines the positive *z* direction along the counter-clockwise beam direction, resulting in a negative pseudorapidity range for detectors like the muon spectrometer. However, due to the symmetry of pp collisions, the rapidity is kept positive when presenting results.
